# A porphyrin pentamer as a bright emitter for NIR OLEDs†

**DOI:** 10.1039/d1tc05951c

**Published:** 2022-03-14

**Authors:** Lara Tejerina, Alexandros G. Rapidis, Michel Rickhaus, Petri Murto, Zewdneh Genene, Ergang Wang, Alessandro Minotto, Harry L. Anderson, Franco Cacialli

**Affiliations:** Chemistry Research Laboratory, Department of Chemistry, University of Oxford Oxford OX1 3TA UK harry.anderson@chem.ox.ac.uk; Department Physics and Astronomy and London Centre for Nanotechnology, University College London London WC1E 6BT UK f.cacialli@ucl.ac.uk; Department of Chemistry and Chemical Engineering/Applied Chemistry, Chalmers University of Technology Gothenburg SE-412 96 Sweden

## Abstract

The luminescence and electroluminescence of an ethyne-linked zinc(ii) porphyrin pentamer have been investigated, by testing blends in two different conjugated polymer matrices, at a range of concentrations. The best results were obtained for blends with the conjugated polymer PIDT-2TPD, at a porphyrin loading of 1 wt%. This host matrix was selected because the excellent overlap between its emission spectrum and the low-energy region of the absorption spectrum of the porphyrin oligomer leads to efficient energy transfer. Thin films of this blend exhibit intense fluorescence in the near-infrared (NIR), with a peak emission wavelength of 886 nm and a photoluminescent quantum yield (PLQY) of 27% in the solid state. Light-emitting diodes (LEDs) fabricated with this blend as the emissive layer achieve average external quantum efficiencies (EQE) of 2.0% with peak emission at 830 nm and a turn-on voltage of 1.6 V. This performance is remarkable for a singlet NIR-emitter; 93% of the photons are emitted in the NIR (*λ* > 700 nm), indicating that conjugated porphyrin oligomers are promising emitters for non-toxic NIR OLEDs.

## Abstract 2

NIR OLEDs are achieved by blending ethyne-linked zinc(II) porphyrin pentamers into a red-emitting push–pull polymeric host matrix (PIDT-2TPD). We report external quantum efficiencies of 2.0 % with peak emission at 830 nm, confirming the promise of porphyrin oligomers for non-toxic NIR OLEDs.

## Graphical abstract

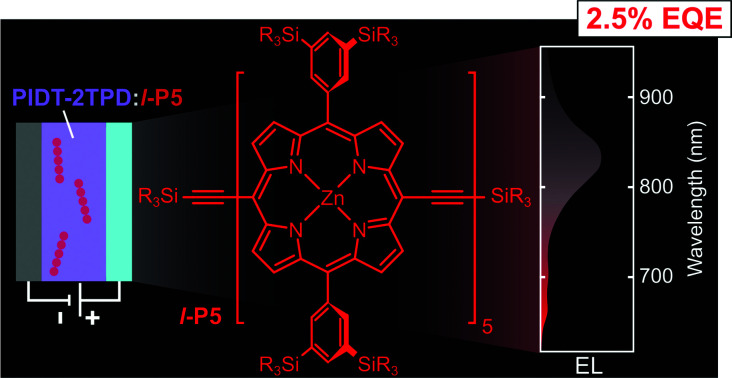

Near infrared (NIR) emitters have a growing diversity of application, spanning from healthcare to optical communication systems. NIR radiation (700 nm < *λ* < 1000 nm) is innocuous to living cells, which in addition to the high transparency of biological tissues at these wavelengths,^1^ makes it useful for imaging, biosensing, and photodynamic therapy.^2–5^ NIR light-emitting diodes (LEDs) have also shown promise for the development of the light-fidelity (Li-Fi) wireless technology,^6,7^ night-vision readable displays and security systems.^8^ Various low-gap chromophores have been applied as emitters in NIR organic light-emitting diodes (OLEDs), including small molecules, conjugated polymers, thermally activated delayed fluorescent (TADF) materials and transition metal complexes.^9^ The HOMO–LUMO gap of an organic molecule can be contracted by extending the π-system, and common synthetic strategies for shifting emission into the NIR include incorporation of conjugated linkages, aromatic substituents, and functional groups having different electronic effects (*e.g.* donor–acceptor combinations). Transition metal complexes with heavy-metal atoms induce spin–orbit coupling leading to efficient phosphorescence,^10^ and their aggregation has been recently leveraged to circumvent the energy-gap law.^11^ Triplet states can also be exploited *via* TADF,^11^ and efficient doublet emitters with electroluminescent spectra partially in the NIR (peaking ∼ 700 nm) have been recently demonstrated.^12^

Porphyrins and related tetrapyrrole macrocycles are versatile chromophores with outstanding and tunable optical properties. Together with their high thermal and photochemical stability, they are widely exploited in natural and artificial light-harvesting systems.^13,14^ Porphyrin-based NIR electroluminescence (EL) has been achieved with phosphorescent platinum(ii) porphyrin monomer complexes,^15–17^ or by connecting fluorescent zinc(ii) porphyrins leading to π-extended wires.^18–21^ In particular, ethyne and butadiyne linkages are effective at narrowing the HOMO–LUMO gap in these conjugated oligomers.^22,23^ The length of the oligomer can be extended by Sonogashira or Glaser–Hay coupling, shifting the emission into the NIR. Alkyne-linked porphyrin oligomers often have higher photoluminescence quantum yields (PLQYs) than the corresponding monomers, because coupling between the porphyrin units results in higher oscillator strengths, accelerating radiative decay, so that it competes more effectively with nonradiative decay channels.^24,25^ These emitters are typically blended with fluorescent host polymers, when incorporated in OLEDs, because they are prone to π-stacking and aggregation, which otherwise tends to quench emission in the solid state.^18,19,21^

Here, we present a linear *meso*-ethyne-linked zinc porphyrin pentamer (*l*-P5, Fig. 1a) which exhibits excellent EL performance in the NIR, when blended with the polymeric hosts poly(9,9-dioctylfluorene-*alt*-benzothiadiazole) (F8BT) and poly[4,4,9,9-tetrakis(4-hexylphenyl)-4,9-dihydro-*s*-indaceno[1,2-*b*:5,6-*b*′]dithiophene-2,7-diyl-*alt*-5,5-bis(2-octyldodecyl)-4*H*,4′*H*-[1,1-bithieno-[3,4-*c*]pyrrole]-4,4,6,6′(5*H*,5′*H*)-tetrone-3,3-diyl] PIDT-2TPD (Fig. 1a). Zinc is a light, non-toxic, earth-abundant transition metal and, importantly, central zinc(ii) confers stability and suitable frontier energy levels to the porphyrins, thus adopting a preferable type-I heterojunction with the polymeric host (Fig. 1), which favors energy transfer over charge transfer.^26^F8BT has been previously used as host matrix in blended NIR OLEDs with porphyrin oligomer guests,^19,21^ and other low-gap emitters.^27,28^ We also investigated the red-emitting push–pull polymer PIDT-2TPD,^26,29^ because its fluorescence spectrum overlaps well with the low-energy region of the absorption spectrum of *l*-P5, whereas F8BT emits in the middle of the visible spectrum (green).

**Fig. 1:**
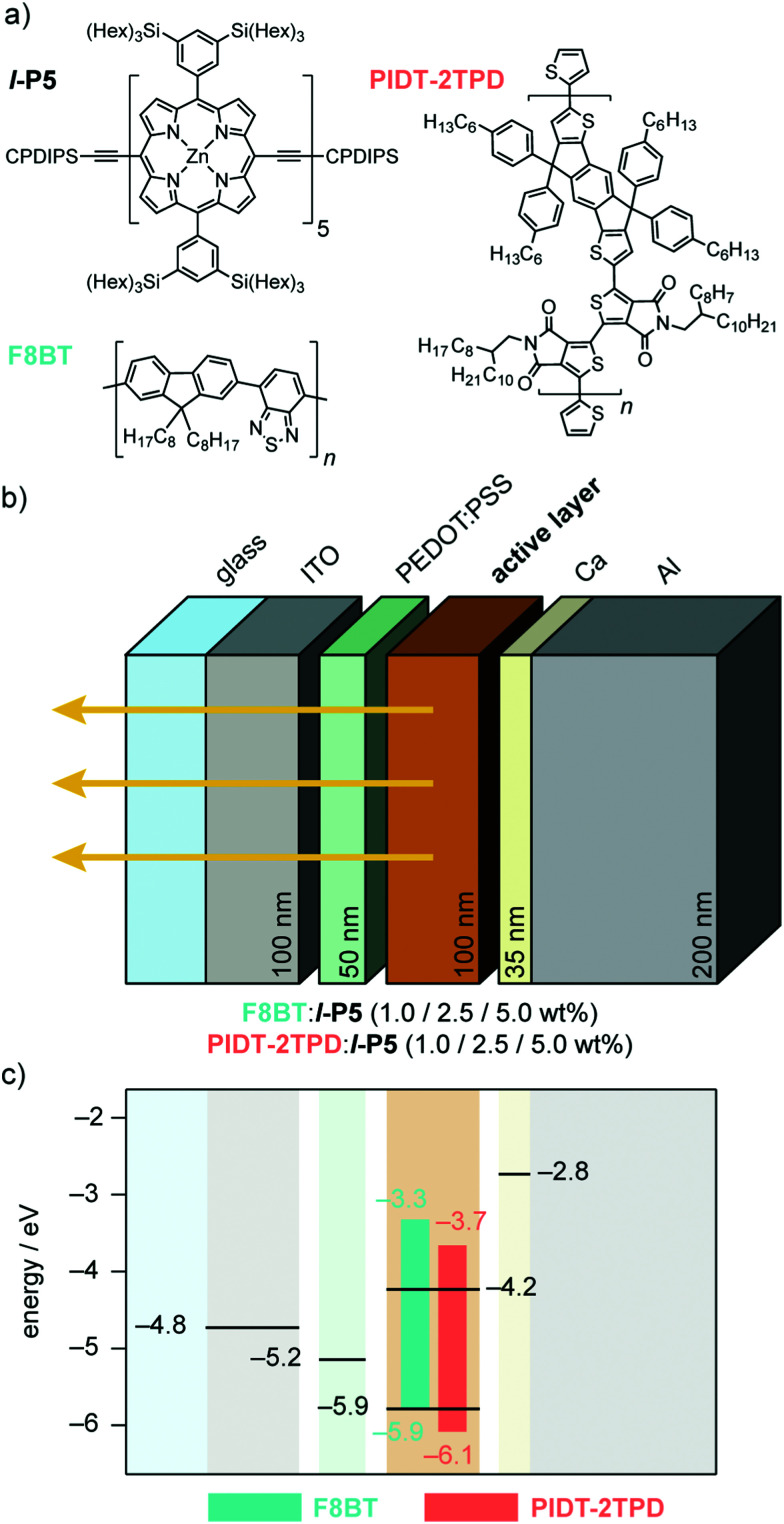
(a) Molecular structures of *l*-P5 porphyrin pentamer, and host polymers F8BT and PIDT-2TPD. CPDIPS = Si(i-Pr)_2_(CH_2_)_3_CN. (b) Multilayer OLED device architecture. From left to right: indium tin oxide (ITO) coated glass anode, poly(3,4-ethylenedioxythiophene) doped with poly(styrene sulfonate) (PEDOT:PSS) hole-transporting layer, blended active layer (F8BT:*l*-P5 or PIDT-2TPD:*l*-P5), and Ca/Al cathode. Layer thicknesses are in nm (c) Schematic band diagram representing the HOMO (bottom of bar) and LUMO (top of bar) energy levels of F8BT and PIDT-2TPD. The energies of the frontier orbitals of *l*-P5 determined electrochemically are represented in black in the emissive layer (see Fig. S1 for electrochemical data, ESI†). The HOMO level of the PEDOT:PSS and the work functions of the electrodes are indicated.

The porphyrin pentamer *l*-P5 was prepared as reported previously with an overall yield of 39% from zinc porphyrin monomer.^30,31^ The ethyne bridges mediate effective π-conjugation,^22^ due to the short inter-porphyrin distance, thus leading to red-shifted absorption and fluorescence spectra, compared with the butadiyne-linked analogues.^21^ Bulky trihexylsilyl (THS) side chains help to prevent aggregation quenching by restricting the intermolecular π–π interactions while providing solubility in most organic solvents,^32^ which is necessary for the fabrication of solution-processed devices. When studied as a dilute solution in toluene, *l*-P5 exhibits a photoluminescence (PL) band at 851 nm (Fig. S2, ESI†) with an average fluorescence lifetime of about 2 ns (Fig. S3, ESI†). The solution PLQY is 0.30 ± 0.01 and the spectral purity is such that >99.9% of photons have *λ* > 700 nm, making it promising as a NIR emitter. The reduced structural flexibility imposed by the short ethyne bridges contributes to a low rate of non-radiative deactivations, while the linear arrangement of the porphyrin units amplifies the rate of radiative decay, thus resulting in exceptionally efficient NIR emission.^18,21,24^

Blended films of *l*-P5 and the polymeric hosts were spin-coated to a thickness of *ca.* 100 nm by using solutions in toluene, with various porphyrin matrix ratios (*i.e.* 1.0, 2.5, 5.0 wt%; see ESI†). The visible absorption spectra of these blends are dominated by the polymer matrix (peaking at either 463 nm for F8BT; or 565 and 612 nm for PIDT-2TPD) with the Q-band of *l*-P5 discernible in the NIR region (Fig. 2a and c). These lowest-energy absorption bands peak at longer wavelengths, compared with *l*-P5 in solution (*ca.* 70 and 45 nm for blends made of F8BT and PIDT-2TPD respectively) which might indicate some planarization of the porphyrin oligomer in the solid blend.^33^ The PL spectra of blends made of PIDT-2TPD (Fig. 2d) show a significantly high fraction of NIR emission (≥95%) for each porphyrin loading, and increasing the loading of the porphyrin pentamer increases the percentage of emission in the NIR region concomitantly with quenching of the matrix emission. This trend is also observed for the PL spectra of blends made of F8BT (Fig. 2b), in which the progressive quenching of the host emission is clearly observed with increasing porphyrin guest loading, however, the highest NIR emission fraction does not exceed 62%. This remarkable difference in the PL spectra between both types of blends is a direct consequence of the poorer spectral overlap between the emission band of the polymeric host F8BT (peaking at *ca.* 550 nm) and the absorption Q-band of the porphyrin pentamer in comparison to the PIDT-2TPD host. The spectral overlap is significantly increased by using the lower-energy emitting PIDT-2TPD host matrix (PL peaking at *ca.* 695 nm, see also Fig. S4 overlapped guest absorption and donors emission spectra, ESI†), resulting in a more efficient host–guest resonant energy transfer. As discussed above for the absorption, the PL of *l*-P5 in the solid-state films is significantly redshifted compared to solutions, with emission bands at *ca.* 919 and 886 nm for blends made of F8BT and PIDT-2TPD respectively, shifting slightly deeper into the NIR with increasing the amount of porphyrin, which we attribute to increasing extents of aggregation. The PL efficiency of *l*-P5 in the PIDT-2TPD-based thin films with high porphyrin loadings (*i.e.* 2.5, 5.0 wt%) is approximately twice that of the F8BT analogues (see legend in Fig. 2b and d). The best performance was achieved by blended films with only 1.0 wt% in *l*-P5, reaching a maximum yield of 0.27 ± 0.02 over the whole spectrum when PIDT-2TPD is used as a host matrix and 0.17 ± 0.04 for the F8BT-based film. It is remarkable that such highly efficient emission is obtained without the need for a disaggregating additive, such as the previously used 4-benzylpyridine.^19^ The PLQY of *l*-P5 in solid films of PIDT-2TPD is almost as high as for the dilute solution in toluene (0.27 *vs.* 0.30). Given that the solid films containing 1.0 and 2.5 wt% of *l*-P5 gave the best trade-off between PLQY and NIR emission fraction for either host polymer, we focused on these two concentrations for testing the EL performance.

**Fig. 2:**
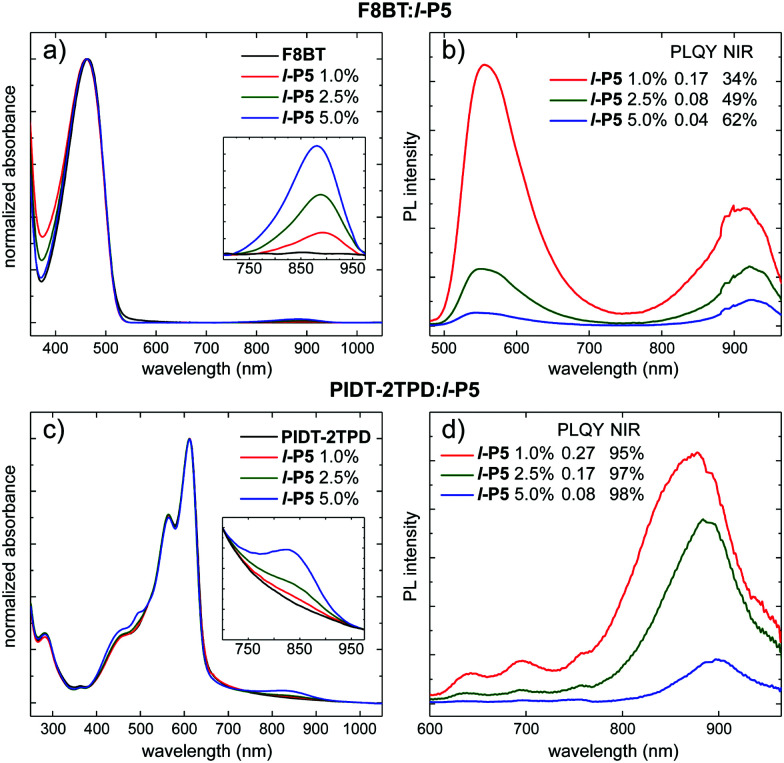
Absorption (a and c) and PL (b and d) spectra of F8BT:*l*-P5 and PIDT-2TPD:*l*-P5 blends in solid-state thin films at different *l*-P5 loadings (1.0, 2.5, and 5.0 wt%). The PL spectra are normalized so that the areas of the peaks are proportional to the PLQYs. PLQY values measured over the whole emission spectrum are reported in the legend along with the fraction emitted in the NIR region (*λ* > 700 nm). PLQY values were measured with an integrated sphere with either a 450 or 520 nm laser diode (in air, at room temperature) for the F8BT:*l*-P5 and PIDT-2TPD:*l*-P5 blends, respectively.

We tested OLEDs with blended light-emitting layers consisting of either F8BT:*l*-P5 or PIDT-2TPD:*l*-P5 at different pentamer concentrations, namely 1.0 and 2.5 wt%, thus making a total of four different types of devices, all fabricated according to the architecture depicted in Fig. 1b. We used an ITO transparent anode, poly(3,4-ethylenedioxythiophene) doped with poly(styrene sulfonate) as a hole-transporting layer, and Ca/Al cathode (see ESI†).^19,21,26,28^ The energies of the frontier orbitals of *l*-P5 (HOMO: −5.9 eV; LUMO: −4.2 eV; Fig. 1c and Fig. S1, ESI†) were measured electrochemically and are nested within the bandgap of the host matrixes, although the measured HOMO level of *l*-P5 lies close to the literature values for F8BT HOMO (*i.e.* −5.9 eV).^19,21,26,28^ Emission from these devices is dominated by an intense component in the NIR that corresponds to the *l*-P5 guest, whereas the emission of either host polymer (*λ* < 700 nm) is largely quenched (Fig. 3a and Fig. S5, ESI†). As shown in Table 1, the fraction of photons emitted beyond 700 nm is higher for devices made from PIDT-2TPD, following the PL trend previously mentioned for the blended solid thin films. It is also noticeable that the greater the loading of *l*-P5 in the active layer (from 1.0 to 2.5 wt%) the larger the NIR emission, reaching the highest fraction of 96%. The predominant EL emission band displays a full width at half maximum of *ca.* 100 nm (Fig. 3a), which is in line with other NIR organic emitters.^27,28,34–38^ and it peaks at 830 nm for devices made of PIDT-2TPD:*l*-P5 1.0 wt% while it is shifted to 874 nm for those containing F8BT:*l*-P5 1.0 wt%, possibly indicating a more planar conformation of *l*-P5 in the latter case. Interestingly, the porphyrin contribution to the EL spectra are noticeably blue-shifted with respect to the PL spectra of the same blends. We propose that this observation is linked to local heating of the chromophores as a result of both Joule heating and exciton energy transfer and subsequent internal conversion processes happening with higher rate than in the surrounding matrices (please see also pages S8 and S9 of the ESI† for a more extended discussion).

**Fig. 3:**
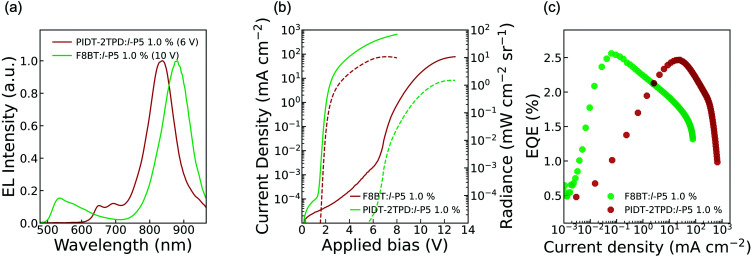
Characteristics of OLEDs incorporating PIDT-2TPD:*l*-P5 (red lines) and F8BT:*l*-P5 (green lines) blends with 1.0 wt% of *l*-P5. (a) EL spectra collected at the maximum radiance voltages indicated in the legend; (b) current density (solid) and radiance (dashed) *vs.* applied bias characteristics; (c) EQE *versus* current density plot.

**Table 1:** OLED performance parameters

Device	*N* a	〈*V*_ON_〉^b^ [V]	〈*R*_MAX_〉^c^ [mW sr^−1^ cm^−2^]	EQE_MAX_^d^ [%]	〈EQE_MAX_〉^e^ [%]	NIR EL^f^ [%]
PIDT-2TPD:*l*-P5 1.0%	5	1.60 ± 0.01	9.43 ± 1.58	2.47	1.98 ± 0.35	93
PIDT-2TPD:*l*-P5 2.5%	6	1.60 ± 0.06	2.09 ± 0.15	1.54	1.40 ± 0.09	96
F8BT:*l*-P5 1.0%	6	4.73 ± 1.25	1.42 ± 0.44	2.56	1.79 ± 0.84	84
F8BT:*l*-P5 2.5%	5	8.70 ± 0.66	0.48 ± 0.19	1.07	0.92 ± 0.11	93

a Number of devices.

b Voltage at which the light output exceeds the noise level, as extrapolated from the *R vs. V* characteristics.

c Average maximum radiance.

d Maximum external quantum efficiency.

e Average external quantum efficiency.

f Photons emitted in the NIR region (*i.e. λ* > 700 nm).

The performance parameters of the OLED devices are summarized in Table 1 and Table ST1 (in ESI†) whereas the current density/radiance *versus* bias voltage (JVR) characteristics and EQE *versus* current density characteristics are shown in Fig. 3b, c and Fig. S6 (ESI†). We also report the electroluminescence spectra collected at the maximum EQE points in Fig. S7 (ESI†). For either polymer matrix, the lowest turn-on voltage (*V*_ON_) and the highest maximum radiance (*R*_MAX_) were obtained for those devices with 1.0 wt% in *l*-P5. Such devices exhibited an average *V*_ON_ of 4.73 ± 1.25 V and 1.60 ± 0.01 V for F8BT and PIDT-2TPD polymer matrixes, respectively. The difference between these values is directly related to the host, and they agree with those reported for similar devices containing different NIR emitting guests, with a smaller HOMO–LUMO (host) gap requiring a smaller *V*_ON_.^26,28^ The maximum radiance (*R*_MAX_) follows the same trend, achieving the highest average value of 9.4 ± 1.6 mW sr^−1^ cm^−2^ for the devices made of PIDT-2TPD:*l*-P5 1.0 wt%, which is remarkably high compared to previously reported NIR organic emitters.^26–28,35,36^ The external quantum efficiency (EQE) is the key parameter for comparing the efficiencies of OLEDs, and it is strongly affected by the device architecture and the intrinsic properties of the emitter,^9^ such as the luminescence efficiency (PLQY). In fluorescent NIR OLED devices, the PLQY is generally limited by two factors: (a) aggregation quenching, and (b) the “energy-gap law”, which predicts a progressively greater vibrational overlapping of the ground and excited states as the energy gap narrows, resulting in increased non-radiative losses.^9^ In particular, with porphyrin fluorescent materials, EQEs ranging from 0.10–1.1% have been reported to date.^18,19,21,39,40^ However, here we obtained the highest average EQE values of 1.8 ± 0.8% and 2.0 ± 0.4% for the devices made of 1.0 wt% of *l*-P5, and F8BT and PIDT-2TPD as host matrixes, respectively. Notably, both emissive layers reached maximum EQEs of 2.5%, measured at current densities of 0.06 mA cm^−2^ (at a driving voltage of 7.1 V) for the F8BT:*l*-P5 1.0 wt%, and 24.9 mA cm^−2^ (at a driving voltage of 3.0 V) for the PIDT-2TPD:*l*-P5 1.0 wt% device, which correspond to optical outputs of 2.3 × 10^−3^ and 0.92 mW sr^−1^ cm^−2^, respectively. The electroluminescence spectra collected at the points of maximum efficiency are reported in Fig. S7 (ESI†).

In summary, the zinc porphyrin pentamer *l*-P5 has demonstrated excellent performance as a NIR emitter in OLEDs. The single-acetylene linkages between porphyrins allow effective π-conjugation throughout the entire oligomer, consequently narrowing the HOMO–LUMO gap, while conferring a molecular rigidity that avoids substantial non-radiative energy losses. Efficient fluorescence in the solid-state thin films was observed beyond 875 nm when *l*-P5 was blended with two different low-energy emitting polymers. NIR electroluminescence was achieved when these blended films were incorporated as an emissive layer in OLEDs. High average EQEs were attained for either host polymer used (2.0% for PIDT-2TPD and 1.8% for F8BT), reaching up to 2.5%. The best performing devices arose from those containing the lowest *l*-P5 loading (1.0 wt%) and the PIDT-2TPD matrix. This seems to be an ideal host–guest match, with PIDT-2TPD accounting for the low *V*_ON_ (1.6 V) and high *R*_MAX_ (9.4 mW sr^−1^ cm^−2^), while *l*-P5 is responsible for the large NIR emission (93 out of 100 photons) upon an efficient resonant energy transfer.

## Conflicts of interest

There are no conflicts to declare.

## Supplementary Material

TC-010-D1TC05951C-s001
