# Supplementary material for: A porphyrin pentamer as a bright emitter for NIR OLEDs
Source: J Mater Chem C Mater. 2022 Mar 14;10(15):5929–33. doi: 10.1039/d1tc05951c (PMC9009301; doi:10.1039/d1tc05951c)
Supplement: TC-010-D1TC05951C-s001 [file TC-010-D1TC05951C-s001.pdf]

Electronic Supplementary Material (ESI) for Journal of Materials Chemistry C.
This journal is © The Royal Society of Chemistry 2022

Electronic Supplementary Information

A Porphyrin Pentamer as a Bright Emitter for NIR OLEDs

Lara Tejerina,[#] Alexandros G. Rapidis,[#] Michel Rickhaus, Petri Murto, Zewdneh Genene, Ergang Wang, Alessandro Minotto, Harry L. Anderson and Franco Cacialli**

[#] These authors contributed equally

Dr. L. Tejerina, Dr. M. Rickhaus, Prof. H. L. Anderson
Chemistry Research Laboratory
Department of Chemistry, University of Oxford
Oxford OX1 3TA, United Kingdom
E-mail: harry.anderson@chem.ox.ac.uk

Dr. Z. Genene, Dr. P. Murto, Prof. E. Wang
Department of Chemistry and Chemical Engineering/Applied Chemistry
Chalmers University of Technology
Gothenburg, SE-412 96, Sweden

A. G. Rapidis, Dr. A. Minotto, Prof. F. Cacialli
Department Physics and Astronomy and London Centre for Nanotechnology
University College London,
London WC1E 6BT, United Kingdom
E-mail: f.cacialli@ucl.ac.uk

Experimental Procedures

Synthesis: The porphyrin pentamer ***l*-P5** was synthesized and characterized as reported previously.[1]

Electrochemistry: Electrochemical experiments were performed using an Autolab PGSTAT12. ***l*-P5** was dissolved in anhydrous CH_2Cl_2 (ca. 0.3 mM) containing Bu_4NBF_4 (0.1 M), under argon. A 3 mm glassy carbon working electrode was used with a Pt wire counter electrode and a Ag/$AgNO_3$ (0.01 M in acetonitrile) reference electrode. The redox potentials were measured using square-wave voltammetry; the reversibility of the redox waves was checked by cyclic voltammetry (Figure S1). Ferrocene (Fc) was used as an internal reference and all the potentials were given relative to the Fc/Fc^+ couple. The first oxidation and reduction potentials (+0.86 V and –0.71 V, *vs.* Fc/Fc^+) were converted to HOMO and LUMO energies applying the equation: $E_{HOMO/LUMO}$ (eV) = –5.1 – $E_{ox/red}$ (*vs.* Fc/Fc^+).[2]

PL characterization: Thin films were spin-casted onto fused silica substrates from 10 mg mL^{-1} toluene solutions. The films were deposited in a N_2 environment via spin-coating at 1500 rpm to obtain a thickness of *ca.* 100 nm, measured with a Dektak profilometer. Photoluminescence was collected from an Andor Shamrock 163 spectrograph coupled with an Andor Newton electron-multiplying charge-coupled device (EMCCD). The PLQY experiments were conducted using an integrating sphere setup, and by comparing the number of photons re-emitted to the number of photons absorbed. Time-resolved PL measurements were carried out with a TCSPC spectrometer previously reported.[3]

OLED characterization: ITO substrates were cleaned with acetone and isopropanol in an ultrasonic bath and treated in an O_2 plasma chamber for 10 min.[4] A 50 nm layer of PEDOT:PSS (Sigma-Aldrich) was spin-coated at 5000 rpm from a 2.8 wt% dispersion in water and annealed at 150 °C for 10 min. The 100 nm active layer was spin-coated on top of the annealed PEDOT:PSS from 10 mg mL^{-1} toluene solutions. A Ca/Al (35/200 nm) cathode was thermally evaporated on top. The samples were then characterized under ca. 10^{-2} mbar vacuum using a Keithley 2400 source meter for both the current measurement and the voltage supply. The optical output of the OLEDs was measured with a calibrated silicon photodiode and the EL spectra were collected with the same (Andor) spectrometer employed for the PL experiments.

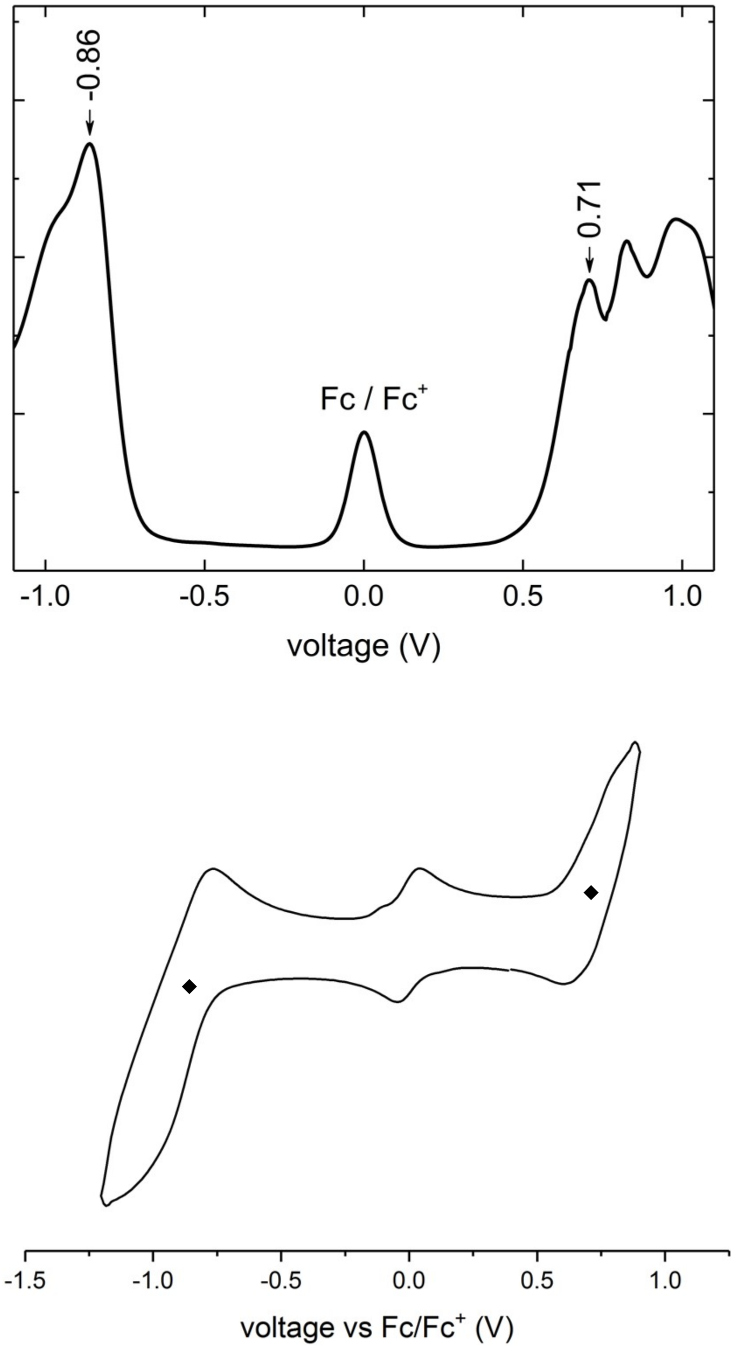

Figure S1. (Top) Square-wave voltammogram (frequency: 5 Hz; step potential: 5 mV; modulation amplitude: 20 mV) and (bottom) cyclic voltammogram (scan rate: 100 mV s^{-1}) of ***l*-P5** in dichloromethane with 0.1 M Bu_4NBF_4. First oxidation and reduction potentials are indicated (+0.71 V and –0.86 V, respectively vs. Fc/Fc^+).

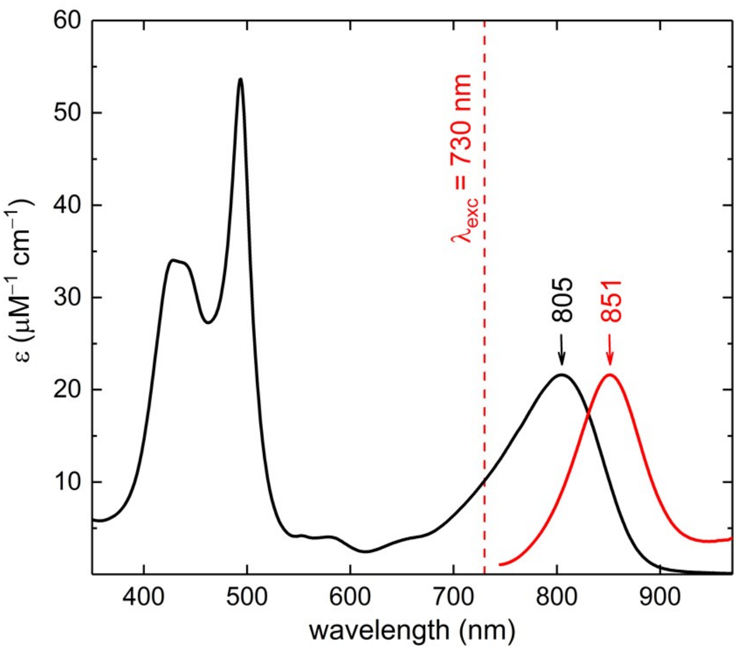

Figure S2. Molar absorption coefficient and PL spectra of the ***l*-P5** porphyrin pentamer in toluene solution. PL was measured at room temperature following excitation at 730 nm. The PL quantum yield is 0.30 ± 0.01.

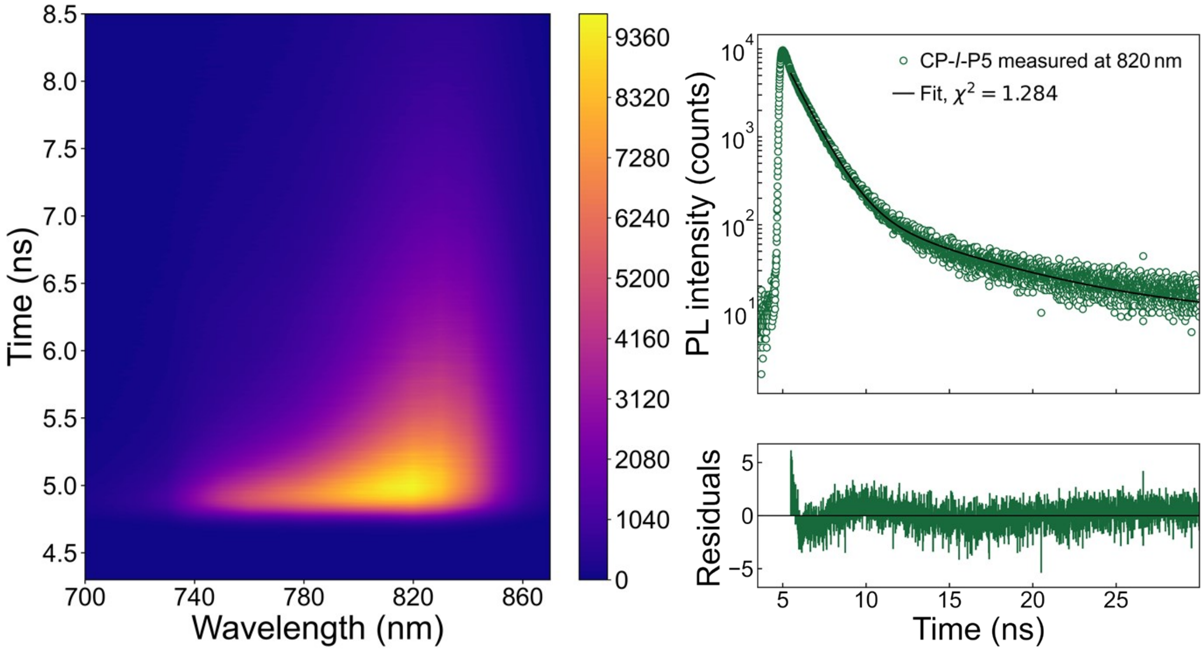

Figure S3. Fluorescence decay time contour plot for ***l*-P5** as a dilute solution in toluene. The decay trace of the emission at 851 nm fit to a biexponential model: the two components are t_1 = 1.15 ns (83%) and t_2 = 6.41 ns (17%). The weighted average of biexponential fits is 2.0 ns.

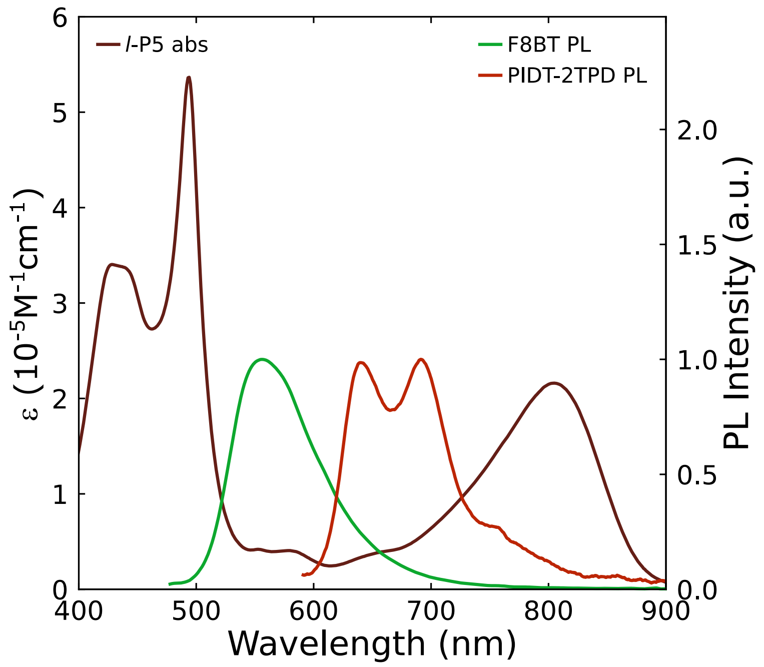

***Figure S4.** Overlapped guest (in solution) absorption and donors (thin films) emission spectra.*

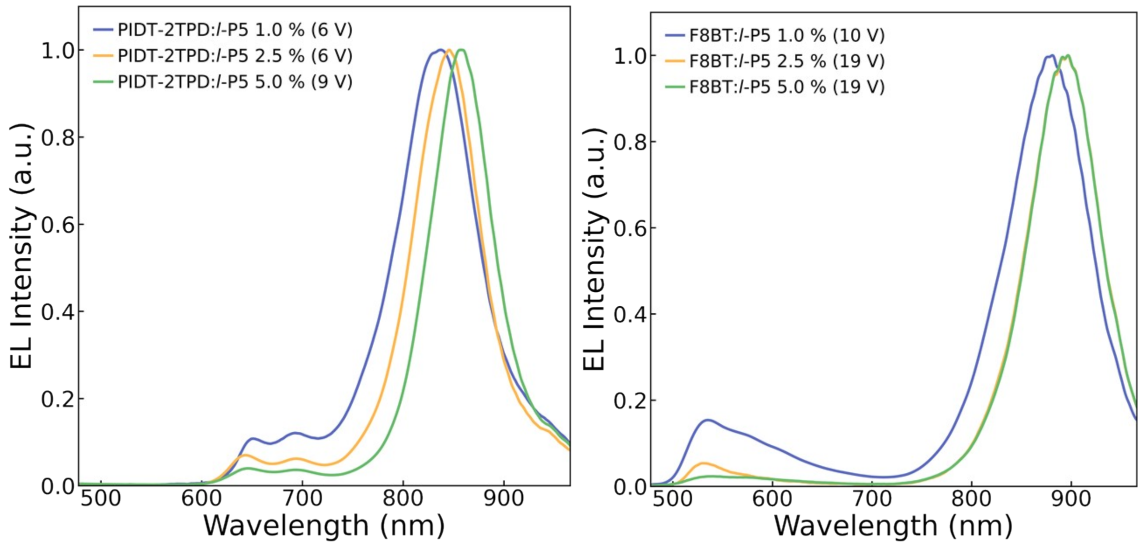

Figure S5. Electroluminescence (EL) spectra collected at the maximum radiance voltages indicated in the legend. Left: **PIDT-2TPD:*l*-P5**; right **F8BT:*l*-P5**.

Table ST1. OLED performance parameters.

Device	*N*[a)]	$<V_{ON}>$[b)] [V]	$<R_{MAX}>$[c)] [mW sr^{-1} cm^{-2}]	EQE_{MAX}[d)] [%]	$<EQE_{MAX}>$[e)] [%]	EL in NIR[f)] [%]
**PIDT-2TPD**	6	1.7	2.3 ± 0.5	1.66	1.55 ± 0.10	46
**PIDT-2TPD:*l*-P5** 1.0%	5	1.60 ± 0.01	9.43 ± 1.58	2.47	1.98 ± 0.35	93
**PIDT-2TPD:*l*-P5** 2.5%	6	1.60 ± 0.06	2.09 ± 0.15	1.54	1.40 ± 0.09	96
**PIDT-2TPD:*l*-P5** 5.0%	6	1.60 ± 0.06	0.99 ± 0.29	1.06	0.73 ± 0.18	95
**F8BT**	6	3.25 ± 0.25	2.90 ± 0.81	0.95	0.77 ± 0.29	5
**F8BT:*l*-P5** 1.0%	6	4.73 ± 1.25	1.42 ± 0.44	2.56	1.79 ± 0.84	84
**F8BT:*l*-P5** 2.5%	5	8.70 ± 0.66	0.48 ± 0.19	1.07	0.92 ± 0.11	93
**F8BT:*l*-P5** 5.0%	4	8.67 ± 0.35	0.25 ± 0.01	0.63	0.56 ± 0.07	97

[a)]number of devices; [b)]voltage at which the light output exceeds the noise level, as extrapolated from the *R vs. V* characteristics; [c)]average maximum radiance; [d)]maximum external quantum efficiency; [e)]average external quantum efficiency; [f)]photons emitted in the NIR region (i.e. λ >700 nm).

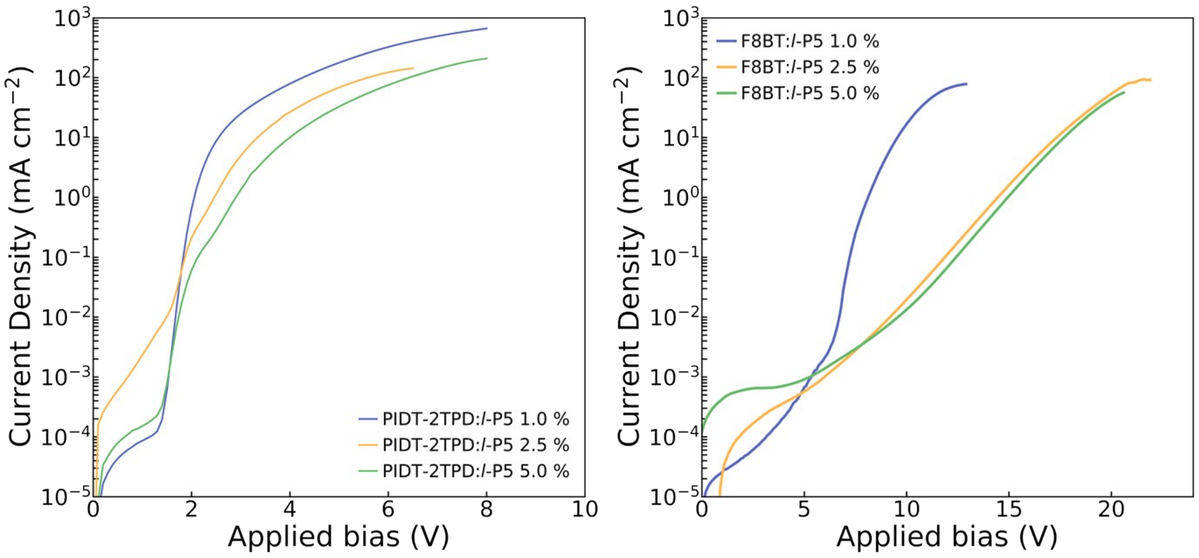

Figure S6. Current density/radiance versus bias voltage (JVR) plots. Current density and radiance are plotted as solid and dotted lines, respectively. Left: **PIDT-2TPD:*l*-P5**; right **F8BT:*l*-P5**.

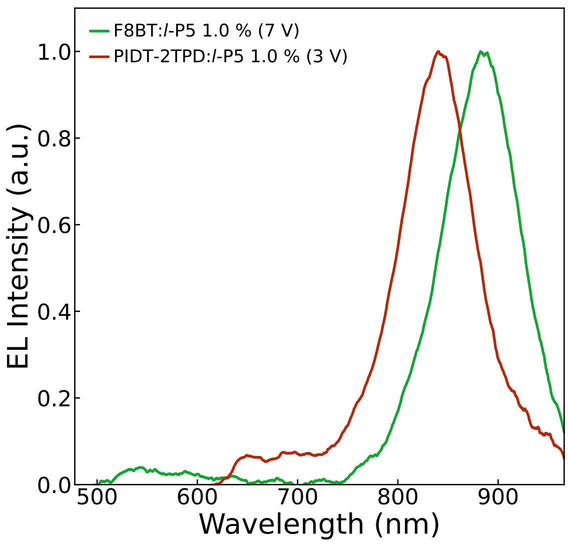

Figure S7. Electroluminescence spectra of representative devices with different blend hosts operated at the points of maximum efficiency (~ 3 V for the PIDT-2TPD host, and ~ 7.1 V for the F8BT host).

On the blueshift of the NIR component of the electroluminescence compared to the photoluminescence of the blends

In general one would expect to observe a slight red-shift of the EL compared to the PL in an organic semiconductor. Such an expectation stems from consideration of the energy-selective nature of charge transport in EL and from the fact that charge transport is extendend trough the whole device thickness (between the electron and the hole of the eventually formed exciton, albeit with a limited cross-section around the charges path). This would favour a more efficient minimization of the exciton energy with respect to the case of spectral migration in a PL experiment, and eventually a red-shift of the EL with respect to the PL, as it is in fact often observed.

However, we note that a blue shift had already been observed in some cases.[5] *In general, a blueshift of either emission or absorption can indicate a reduction of planarity of the emitters which may in turn derive from geometric constraints or environmental factors, such as the temperature. Significantly, thermochromism of organic semiconductors has been reported extensively and also been used as a probe for local heating.*[6] *In addition to heating due to the current flowing through the semiconductors (Joule effect), it is possible that thermalization of "hot" excitons (either formed or transferred onto* ***l-P5****) via internal conversion could affect the local chromophore temperature and thus the spectral position of the emission. Interestingly, the* ***F8BT*** *contribution to the EL spectra of blend devices is also slightly blue-shifted with respect to that of the PL in Fig. 2 (by few nm), thereby suggesting partial heating of this host as well. The blue-shift of the* ***PIDT-2TP*** *contribution to the EL of* ***PIDT-2TP*** *blend is less significant, but we note that these devices generally operate at a lower voltage and that more substantial overlap of the host and guest emission spectra in* ***PIDT-2TP:l-P5*** *devices makes the determination of any spectral shift less straightforward.*

Nevertheless, it is reasonable to expect that the more substantial blue-shift of the porphyrin EL contribution with respect to either matrix can be due to a different thermochromic

sensitivity of the porphyrins with respect to the matrices, or to the fact that, on average and in each time interval, each ***l-P5*** *chromophore is excited many more times than host chromophores, both because of Förster and Dexter transfer from the surrounding matrix, which effectively funnels the available excitonic energy onto the porphyrins, and because porphyrins also act as charge traps and become an elective location for direct exciton formation. The observed blueshift would thus be entirely consistent (and expected) with heating of the chromophores via phonon emission occurring during the frequent internal conversion events necessary to take the excitons to the lowest vibrational state of the excited porphyrin singlets. A lower temperature of the chromophores contributing to the host emission in the blend EL spectra is also expected since these chromophores would be spatially separated by at least a Förster radius from any porphyrin chromophores, also contributing to explain the different shift for the matrices emissions in Fig. 3a. The progressive red-shift of the porphyrin emission peak with increasing concentration of the* ***PIDT-2TPD:l-P5*** *devices (Fig. S5) would also be expected in this scenario, as the average rate of excitation of each NIR chromophore would decrease with increasing concentration. However, increasing aggregation would also contribute to the red-shift thereby making the disentangling of these two effects extremely difficult. Last but not least, progressively more significant chromophore heating at higher currents would also explain the efficiency roll-off typically observed in our devices.*

References

1. R. Haver, L. Tejerina, H. W. Jiang, M. Rickhaus, M. Jirasek, I. Grubner, H. J. Eggimann, L. M. Herz and H. L. Anderson, *J. Am. Chem. Soc.*, 2019, **141**, 7965-7971.
2. C. M. Cardona, W. Li, A. E. Kaifer, D. Stockdale and G. C. Bazan, *Adv. Mater.*, 2011, **23**, 2367-2371.
3. A. Petrozza, S. Brovelli, J. J. Michels, H. L. Anderson, R. H. Friend, C. Silva and F. Cacialli, *Adv. Mat.*, 2008, **20**, 3218-3223.
4. T. M. Brown, G. M. Lazzerini, L. J. Parrott, V. Bodrozic, L. Burgi and F. Cacialli, *Organic Electronics*, 2011, **12**, 623-633.
5. *S. Baysec, A. Minotto, P. Klein, S. Poddi, A. Zampetti, S. Allard, F. Cacialli and U. Scherf, Sci. China-Chem., 2018, 61, 932-939.*
6. *G. Latini, A. Downes, O. Fenwick, A. Ambrosio, M. Allegrini, C. Daniel, C. Silva, P. G. Gucciardi, S. Patane, R. Daik, W. J. Feast and F. Cacialli, Appl. Phys. Lett., 2005,* ***86****, 011102.*
